# Supplementary material for: Resolving the full spectrum of human genome variation using Linked-Reads
Source: Genome Res. 2019 Apr;29(4):635–45. doi: 10.1101/gr.234443.118 (PMC6442396; doi:10.1101/gr.234443.118)
Supplement: Supplemental Material [file supp_gr.234443.118_Supplemental_longranger-2.2.zip › longranger-2.2.tar.bz2!/lr2.2-public/lib/stan/cmdstan/stan/doc/api/html/classstan_1_1agrad_1_1op__dv__vari.html]

Stan: stan::agrad::op\_dv\_vari Class Reference


|  |  |
| --- | --- |
|  | Stan  2.5.0  probability, sampling & optimization |


- Main Page
- Related Pages
- Namespaces
- Classes
- Files

- Class List
- Class Index
- Class Hierarchy
- Class Members

All Classes Namespaces Files Functions Variables Typedefs Enumerations Enumerator Friends Macros Pages

- stan
- agrad
- op\_dv\_vari

Public Member Functions |
Protected Attributes |
List of all members

stan::agrad::op\_dv\_vari Class Reference

`#include <dv_vari.hpp>`

Inheritance diagram for stan::agrad::op\_dv\_vari:

|  |  |
| --- | --- |
| Public Member Functions | |
|  | op\_dv\_vari (double f, double a, vari \*bvi) |
|  | |
| Public Member Functions inherited from stan::agrad::vari | |
|  | vari (const double x) |
|  | Construct a variable implementation from a value. More... |
|  | |
|  | vari (const double x, bool stacked) |
|  | |
| virtual | ~vari () |
|  | Throw an illegal argument exception. More... |
|  | |
| virtual void | init\_dependent () |
|  | Initialize the adjoint for this (dependent) variable to 1. More... |
|  | |
| virtual void | set\_zero\_adjoint () |
|  | Set the adjoint value of this variable to 0. More... |
|  | |
| Public Member Functions inherited from stan::agrad::chainable | |
|  | chainable () |
|  | Construct a chainable object. More... |
|  | |
| virtual | ~chainable () |
|  | Chainables are not destructible and should go on the function call stack or be allocated with operator new. More... |
|  | |
| virtual void | chain () |
|  | Apply the chain rule to this variable based on the variables on which it depends. More... |
|  | |

|  |  |
| --- | --- |
| Protected Attributes | |
| double | ad\_ |
|  | |
| vari \* | bvi\_ |
|  | |

|  |  |
| --- | --- |
| Additional Inherited Members | |
| Static Public Member Functions inherited from stan::agrad::chainable | |
| static void \* | operator new (size\_t nbytes) |
|  | Allocate memory from the underlying memory pool. More... |
|  | |
| static void | operator delete (void \*) |
|  | Delete a pointer from the underlying memory pool. More... |
|  | |
| Public Attributes inherited from stan::agrad::vari | |
| const double | val\_ |
|  | The value of this variable. More... |
|  | |
| double | adj\_ |
|  | The adjoint of this variable, which is the partial derivative of this variable with respect to the root variable. More... |
|  | |

## Detailed Description

Definition at line 9 of file dv\_vari.hpp.

## Constructor & Destructor Documentation

|  |  |  |  |
| --- | --- | --- | --- |
| stan::agrad::op\_dv\_vari::op\_dv\_vari | ( | double | *f*, |
|  |  | double | *a*, |
|  |  | vari \* | *bvi* |
|  | ) |  |  |

inline

Definition at line 14 of file dv\_vari.hpp.

## Member Data Documentation

double stan::agrad::op\_dv\_vari::ad\_

protected

Definition at line 11 of file dv\_vari.hpp.

vari\* stan::agrad::op\_dv\_vari::bvi\_

protected

Definition at line 12 of file dv\_vari.hpp.

---

The documentation for this class was generated from the following file:

- src/stan/agrad/rev/internal/dv\_vari.hpp


---

[ Stan Home Page ]

*© 2011–2014,
Stan Development Team.*
